# Hodgkin's Disease

**DOI:** 10.1038/bjc.1973.10

**Published:** 1973-01

**Authors:** K. A. Newton, D. H. Mackenzie, Margaret F. Spittle, Anna Mikolajczuk

## Abstract

Two hundred and fifty cases of histologically proven Hodgkin's disease have been reviewed. These cases were classified according to the Rye Conference histological classification (Lukes *et al.,* 1966a) and according to the Cross classification (Cross, 1969). Overall, both classifications were reasonably effective in predicting prognosis but that of Cross with its seven sub-groups proved more difficult to use than the simpler Rye classification. In all cases the follow-up period exceeded 5 years. A study was made of the influence of clinical symptoms on survival with particular reference to night sweats, fever, pruritus, anorexia, lassitude, weight loss, haematological abnormalities and splenic enlargement. The presence of these abnormalities adversely affected prognosis. The spread of the disease from one group of nodes to the next was also documented. Considering all cases the 5-year survival was 54%. The 5-year survivals according to histological type were: lymphocytic predominance 69%, nodular sclerosis 57%, mixed cellularity 41%, lymphocytic depletion 40%. The 10-year survival was 23% which, when corrected by the actuarial method (Berkson and Gage, 1950), rose to 36%. The importance of symptomatology as well as histological grading in the prognosis of Hodgkin's disease is confirmed.


*Br. J. Cancer* (1973) **27**, 80

### HODGKIN'S DISEASE

### A CLINICO-PATHOLOGICAL STUDY OF 250 CASES WITH A 5-YEAR FOLLOW-UP

K. A. NEWTON, D. H. MACKENZIE, MARGARET F. SPITTLE AND ANNA MIKOLAJCZUK

*From Westminster Hospital, London, S.W.1.*

Received 11 August 1972. Accepted 4 October 1972

**Summary.—Two hundred and fifty cases of histologically proven Hodgkin's disease have been reviewed. These cases were classified according to the Rye Conference histological classification (Lukes *et al.*, 1966a) and according to the Cross classification (Cross, 1969). Overall, both classifications were reasonably effective in predicting prognosis but that of Cross with its seven sub-groups proved more difficult to use than the simpler Rye classification. In all cases the follow-up period exceeded 5 years. A study was made of the influence of clinical symptoms on survival with particular reference to night sweats, fever, pruritus, anorexia, lassitude, weight loss, haematological abnormalities and splenic enlargement. The presence of these abnormalities adversely affected prognosis. The spread of the disease from one group of nodes to the next was also documented. Considering all cases the 5-year survival was 54%. The 5-year survivals according to histological type were: lymphocytic predominance 69%, nodular sclerosis 57%, mixed cellularity 41%, lymphocytic depletion 40%. The 10-year survival was 23% which, when corrected by the actuarial method (Berkson and Gage, 1950), rose to 36%. The importance of symptomatology as well as histological grading in the prognosis of Hodgkin's disease is confirmed.**

IN 1957 a review of 602 cases of primary tumours of lymphoid tissue was presented from Westminster Hospital (Lumb and Newton, 1957). In recent years there have been a number of advances in the classification and treatment of certain lymphomata. The present communication deals exclusively with cases of Hodgkin's disease and these have been considered from the points of view of histology and clinical findings in relation to prognosis.

### PART I—PATHOLOGY

Adequate histological sections were available in all the 250 cases presented here and these were reviewed by one of us (D.H.M.) without knowledge of the clinical histories. On histological grounds they were classified according to the Rye Conference histological classification (Lukes *et al.*, 1966*a*) and according to the Cross classification (Cross, 1969). Only the Rye classification will be used in this communication. The classification of Cross will form the subject of a separate communication. In an appreciable number of cases the poor quality of the sections prevented classification and these have been excluded from the present study.

First, certain general points need emphasis. While the majority of cases in any series will fall fairly readily into one of the sub-divisions, borderline and debatable cases are unavoidable. The degree to which experienced pathologists may differ has been clearly demonstrated recently (Keller *et al.*, 1968). Secondly, slides which may be described as adequate rather than excellent may contain enough distortion to render the identification of lacuna cells difficult. The Cross classification with its greater emphasis on cytological detail in the assessment of differentiation is particularly dependent on excellent preparations. Lastly the tendency of surgeons to remove small and easily accessible nodes from an affected group may present a pathologist with material unrepresentative of the disease as a whole.

The older classifications of Hodgkin's disease regardless of terminology suffered from one great defect. The recognition of a relatively benign form and a particularly aggressive one still left over 80% in a single group with a very wide spectrum of survival time. Recent classifications (Lukes *et al.*, 1966*a*; Lukes, Butler and Hicks, 1966*b*; Cross, 1969) have endeavoured to split this 80% into sub-groups in which a histological picture is more closely related to prognosis. An ideal classification would be intelligible to and useable by pathologists who do not claim to have made a special study of lymph nodes. It should also give a reasonable guide to prognosis.

The classification of Lukes and his co-workers (1966*a*; 1966*b*) is well known and will not be described here. The so-called Rye classification (Lukes *et al.*, 1966*a*) is a simplified form of the original one and divides Hodgkin's disease into four separate histological types: 1. lymphocytic predominance; 2. nodular sclerosis; 3. mixed cellularity, and 4. lymphocytic depletion. The term lymphocytic predominance is used to include the lymphocytic and/or histiocytic types both nodular and diffuse, of the original Lukes classification. The term lymphocytic depletion includes the diffuse fibrosis and reticular types of the same classification.

A comment about nodular sclerosing Hodgkin's disease is necessary. The incidence of this particular type has varied widely from series to series, *e.g.* Kadin, Glatstein and Dorfman (1971) 73%, Keller *et al.* (1968) 52%, Franssila, Kalima and Voutilainen (1967) 47%, Lukes *et al.* (1966*b*) 40%, Cross (1968) 16%. This is due in part to a certain vagueness of definition, particularly in respect of the amount of collagen necessary to justify the diagnosis and whether the presence of the lacuna form of Reed-Sternberg cell is an absolute prerequisite. In addition some workers (Kadin *et al.*, 1971; Strüm and Rappaport, 1971) accept the concept of a cellular phase of nodular sclerosis where lacuna cells are present without any collagen. In the present series we have included under nodular sclerosis those nodes where islands of Hodgkin tissue were quite clearly demarcated by broad bands of collagen. Lacuna cells were present in the great majority of cases. In a very few instances they were not identified with certainty but these cases have been included if the fibrotic element was characteristic.

The distribution of the 250 cases in the present series between the sub-groups is shown in Table I.

TABLE I.—*Rye Classification. Distribution of Histological Types*

| | Franssila *et al.* | Keller *et al.* | Present series | Lukes *et al.* |
|---|---|---|---|---|
| Lymphocytic predominance | 9% | 5% | 29% | 16% |
| Nodular sclerosis | 47% | 52% | 31% | 40% |
| Mixed cellularity | 33% | 37% | 24% | 26% |
| Lymphocytic depletion | 11% | 6% | 16% | 18% |

They are compared with the figures found in other series (Franssila *et al.*, 1967; Keller *et al.*, 1968). The figures obtained by Lukes *et al.* (1966*b*) are also shown translated into the Rye terminology.

*Relation of survival to histological type*

All 250 cases were followed for at least 5 years. Fig. 1–3 show the percentage survivals for all cases; for cases where there was no record in the notes of night sweats, pruritus or fever, and for cases with no systemic symptoms and only one group of nodes involved on presentation. After 5 years the number available for study slowly declined, but the 10-year survival figure for all cases was 23% which, when corrected by the actuarial method, rose to 36%.

A study of Fig. 1, 2 and 3 shows both expected and unexpected findings. For all cases (Fig. 1) the survivals are as expected although the difference between the mixed cellularity and lymphocytic depletion groups is negligible at 5 years. With patients with no systemic symptoms the lymphocytic depletion group did better than the mixed cellularity one. Unexpectedly long survivals of patients with anaplastic tumours were noted in a previous series reported from this hospital (Lumb and Newton, 1957). For patients with no systemic effects and only one group of nodes involved (Fig. 3) the same relationship was noted between the mixed cellularity and lymphocytic depletion groups, but unexpectedly, the nodular sclerosis group did worst of all. As these patients were not subjected to a diagnostic laparotomy the staging does not take fully into account the possible presence of occult disease below the diaphragm. As

FIG. 1.—Relation of survival to histologic type. All cases. Rye classification.

FIG. 2.—Relation of survival to histological type for cases with no night sweats, pruritus or fever. Rye classification.

FIG. 3.—Relation of survival to histological type for cases with no night sweats, pruritus or fever and only one group of nodes involved. Rye classification.

the majority of nodular sclerosis Stage I patients had disease in the cervical areas only and not in the mediastinum, and noting the converse relationship found by Glatstein *et al.* (1970) between mediastinal and abdominal disease, it may be that the relatively poor survival in early stage nodular sclerosis was due to undetected para-aortic disease.

### PART II—CLINICAL CONSIDERATIONS

These 250 cases were analysed with the assistance of the Westminster Hospital Medical Computer Centre. The relationships between symptoms and survival, and symptoms and histology were evaluated. A study was also made of nodal involvement and of the spread of the disease from one group of nodes to another. In this section only the Rye histological classification is used. In some cases documentation was inadequate in respect of certain clinical details, an unfortunate hazard of retrospective surveys. However, details of the disease process were adequate and all the patients had been followed for 5 years.

The staging criteria used in this study were those of Rosenberg *et al.* (1971). As this retrospective series contained no extra nodal lymphomata and laparotomies were not performed, the sub-staging used for such cases in this classification has been omitted from Table II.

TABLE II.—*Clinical Classification*

| Stage | Criteria |
|---|---|
| I . | Involvement of a single lymph node region. |
| II . | Involvement of two or more lymph node regions on the same side of the diaphragm. |
| III . | Involvement of lymph node regions on both sides of the diaphragm which may be accompanied by splenic involvement. |
| IV . | Diffuse or disseminated involvement of one or more extra lymphatic organs or tissues with or without associated lymph node enlargement. |
| | The sub-staging of all patients with extra lymphatic or splenic involvement initially has not been used as this was not applicable to the series described. |

All stages were sub-classified " A " or " B " to indicate the absence or presence respectively of systemic symptoms—pruritus, night sweats or otherwise unexpected fever. A single elevated temperature reading was not thought significant enough to classify the patient in the " B " group. The staging of this series is shown in Table III.

The present study attempts to evaluate other symptoms not considered sufficient in themselves to relegate a patient into the " B " sub-group by this Committee. Lymphangiography and mediastinal tomography were not in use as diagnostic aids

TABLE III.—*Clinical Staging of Cases*

| Stage | Number of cases |
|---|---|
| I A | 111 |
| I B | 32 |
| II A | 64 |
| II B | 17 |
| III A | 11 |
| III B | 13 |
| IV A | 2 |
| IV B | 0 |
| Total | 250 |

when the majority of these patients first presented and the staging of the disease may be inaccurate in this respect. This probably explains the high number of Stage I cases.

*Survival time*

Besides the actuarial method (Berkson and Gage, 1950) of expressing survival rate, the mean and median survival times are used. For these the survival time is taken to be the time from onset of disease to death, or to the time of the survey in the case of live patients. The mean is the average of the survival times, and the median is the survival time of the patient in the middle of the series when the patients are arranged in order according to survival time. The main advantage of the median is that, provided it is less than the 5-year follow-up period, it will remain unaffected by patients who are still alive. However, in a number of cases the median is greater than 5 years so both mean and median are used where appropriate.

*Age and sex*

The age incidence is shown in Fig. 4. The peak incidence was the third decade and the age range was 7–78 years. The mean survival time decreased with age (Fig. 5) except in the 70–80 year group where there were only 3 patients. This decrease was even more striking in the male population (Fig. 6). These findings are in agreement with Peters and Middlemiss (1958) but at variance with Easson (1966) who found that in males the survival rate was not influenced by age.

FIG. 4.—Distribution of series by age.

FIG. 5.—Relationship of mean survival to age.

FIG. 6.—Relationship of mean survival to age and sex.

Females over the age of 55 had a strikingly poorer 5-year survival than women under 55. When compared with other series (Johnson *et al.*, 1970; Peters and Middlemiss, 1958) there was an unusually high preponderance of males (3 : 1). This can be accounted for by the inclusion of patients referred by the Armed Forces.

Women of all age groups lived as long or longer than men (Fig. 6). This may be due to the fact that the nodular sclerosing type of disease occurred in 44% of female patients as against 27% of males. The fact that no significant difference in survival time could be found between the sexes for any particular histological group supports this view. The distribution of males and females in the histological sub-groups is shown in Table IV.

*Influence of symptoms on survival*

In addition to the classic trio of night sweats, pruritus and fever, further presenting clinical features were studied to determine their influence on survival. In assessing the different criteria listed in Table V it should be made clear that there are three categories: yes—denoting the presence of the feature; no—denoting its absence, and N.I.—when this factor was not indicated in the notes.

The lack of precise information regarding certain signs and symptoms in an appreciable number of cases is unfortunate, but is an inherent problem in any large retrospective survey.

It can be seen from Table V that anorexia, feeling unwell, lassitude, night sweats and weight loss reduced the survival time. Other features which significantly reduced survival were a haemoglobin below 11·4 g %, a white cell count above 15,000 per mm$^3$ and a temperature of 37·2° C or above. These findings may be contrasted with those of Fuller *et al.* (1971) who found no significant reduction in survival time in patients with constitutional symptoms. Features which made no significant difference to survival were alcohol intolerance, cough and dyspnoea. Pruritus, long accepted as a criterion for Stage B disease, made no significant difference to survival time in this series when the cases were considered collectively regardless of histology. This is in agreement with the new international classification. However, when the histological sub-groups are considered separately, the presence of pruritus in the lymphocytic predominant group adversely affected prognosis to a significant degree ($0·05 < P < 0·10$) (Table VI).

*Relation of symptoms to histology*

The numbers in each individual group are relatively small and it is difficult to assess their statistical significance. The findings are shown in Table VII and the following comments may be made.

1. *Feeling unwell and lassitude.*—These features were commonest in the lymphocytic depletion group.
2. *Night sweats.*—The highest percentage of night sweats occurred in the nodular sclerosis group.
3. *Pruritus.*—This symptom was most frequent in the nodular sclerosis group (51%), and lowest with lymphocytic depletion (23%).
4. *Alcohol intolerance.*—This was commonest in the mixed cellularity variety and was not recorded at all with lymphocytic depletion.
5. *Cough.*—This occurred most commonly with nodular sclerosis (35%). This is in keeping with the predilection of this histological variety for the mediastinum.
6. *Abnormal blood counts.*—A haemoglobin level less than 11·4 g % was found

TABLE IV.—*Relation Between Sex and Histological Group*

| | Lymphocytic predominance | Nodular sclerosis | Mixed cellularity | Lymphocytic depletion |
|---|---|---|---|---|
| Males | 61 | 51 | 49 | 30 |
| Females | 11 | 26 | 13 | 9 |
| Total | 72 | 77 | 62 | 39 |
| % Males | 32 | 27 | 26 | 15 |
| % Females | 18 | 44 | 22 | 16 |

The difference in distribution of histology between the sexes is significant ($0·05 < P < 0·1$).

TABLE V.—*Relationship of Survival Time to Signs, Symptoms and Simple Investigations*

| | | Numbers | Survival in months: Mean | Median | Level of sig. $P$ |
|---|---|---|---|---|---|
| Well | Yes | 119 | 83 | 72 | $P < 0{\cdot}001$ |
| | No | 121 | 51 | 44 | |
| | NI | 10 | | | |
| Lassitude | Yes | 125 | 51 | 47 | $P < 0{\cdot}001$ |
| | No | 109 | 83 | 72 | |
| | NI | 16 | | | |
| Night sweats | Yes | 56 | 41 | 34 | $P < 0{\cdot}005$ |
| | No | 58 | 78 | 72 | |
| | NI | 136 | | | |
| Pruritus | Yes | 47 | 62 | 57 | NSD* |
| | No | 69 | 63 | 60 | |
| | NI | 134 | | | |
| Cough | Yes | 45 | 53 | 52 | NSD* |
| | No | 142 | 68 | 60 | |
| | NI | 63 | | | |
| Dyspnoea | Yes | 24 | 50 | 39 | NSD* |
| | No | 151 | 66 | 60 | |
| | NI | 75 | | | |
| Anorexia | Yes | 36 | 44 | 41 | $0{\cdot}025 < P < 0{\cdot}05$ |
| | No | 145 | 68 | 60 | |
| | NI | 69 | | | |
| Weight loss | Yes | 71 | 44 | 28 | $P < 0{\cdot}001$ |
| | No | 119 | 80 | 72 | |
| | NI | 60 | | | |
| Alcohol intolerance | Yes | 11 | 51 | 34 | NSD* |
| | No | 71 | 61 | 60 | |
| | NI | 168 | | | |
| W.B.C. 15,000 | Above | 14 | 46 | 47 | $P < 0{\cdot}1$ |
| | Below | 159 | 67 | 60 | |
| | NI | 77 | | | |
| Hb. 11·4 g | Above | 134 | 69 | 60 | $P < 0{\cdot}05$ |
| | Below | 43 | 45 | 34 | |
| | NI | 73 | | | |
| Temperature 37·2° C | Above | 75 | 49 | 34 | $P < 0{\cdot}025$ |
| | Below | 49 | 70 | 60 | |
| | NI | 126 | | | |

* No significant difference.

in 41% of patients with lymphocytic depletion. A white blood count in excess of 15,000 per mm³ was found in 13% of nodular sclerosis cases and in 10% of the lymphocytic depletion ones.

7. *Fever.*—A temperature of 37·2° C or above on presentation was found in 82% of the lymphocytic depletion cases. It should be emphasized that the temperature figures refer to an isolated reading on presentation and do not imply a prolonged pyrexia or Pel-Epstein type fever.

8. *Splenomegaly.*—The incidence of splenomegaly was assessed (a) on presentation and (b) as a finding during the progress of the disease. There was no significant difference in the survival of patients who developed a palpable spleen during the course of the disease and those with no splenomegaly. However, the presence of splenomegaly on presentation had a significant effect ($P < 0{\cdot}001$) on survival. Thus the median survival time for 24 patients presenting with splenomegaly was 17 months, whereas for 226 patients with no splenomegaly on presentation it was 60 months.

When used as a guide to prognosis it is interesting to note the differences in

TABLE VI.—*Median Survival Time (in Months) Related to Symptoms for Each Histological Group*

| Rye group | | Lymphocytic predominance | Nodular sclerosis | Mixed cellularity | Lymphocytic depletion |
|---|---|---|---|---|---|
| Well | Yes | 84 | 83 | 48 | 60 |
| | No | 44 | 56 | 38 | 26 |
| Lassitude | Yes | 44 | 56 | 38 | 26 |
| | No | 84 | 83 | 50 | 60 |
| Night sweats | Yes | 34 | 52 | 21 | 15 |
| | No | 72 | 78 | 55 | 39 |
| Pruritus | Yes | 47 | 60 | 42 | 37 |
| | No | 65 | 63 | 35 | 60 |
| Cough | Yes | 60 | 61 | 26 | 29 |
| | No | 72 | 60 | 50 | 38 |
| Dyspnoea | Yes | 60 | 60 | 27 | 19 |
| | No | 72 | 63 | 47 | 46 |
| Anorexia | Yes | 29 | 60 | 24 | 17 |
| | No | 72 | 65 | 35 | 38 |
| Weight loss | Yes | 28 | 55 | 25 | 17 |
| | No | 84 | 84 | 48 | 54 |
| Alcohol intolerance | Yes | 8 | 34 | 67 | 0 |
| | No | 60 | 57 | 25 | 32 |
| WBC $\geqslant$ 15,000 | Yes | 66 | 34 | 49 | 17 |
| | No | 72 | 60 | 50 | 35 |
| Hb. < 11·4 g. | Yes | 72 | 45 | 16 | 24 |
| | No | 70 | 62 | 60 | 29 |
| T° $\geqslant$ 37·2° C | Yes | 60 | 44 | 27 | 17 |
| | No | 70 | 65 | 42 | 53 |

TABLE VII.—*Numbers of Patients Grouped According to Rye Histology and Symptoms and Simple Clinical Criteria*

| | | Lymphocytic predominance | Nodular sclerosis | Mixed cellularity | Lymphocytic depletion |
|---|---|---|---|---|---|
| Well | Yes | 39 (57%) | 34 (47%) | 32 (53%) | 14 (37%) |
| | No | 30 | 39 | 28 | 24 |
| Lassitude | Yes | 32 (47%) | 41 (58%) | 28 (49%) | 24 (63%) |
| | No | 36 | 30 | 29 | 14 |
| Night sweats | Yes | 10 (30%) | 27 (61%) | 9 (47%) | 10 (53%) |
| | No | 23 | 17 | 10 | 8 |
| Pruritus | Yes | 13 (36%) | 19 (51%) | 11 (42%) | 4 (23%) |
| | No | 23 | 18 | 15 | 13 |
| Cough | Yes | 9 (17%) | 20 (35%) | 9 (19%) | 7 (23%) |
| | No | 44 | 38 | 37 | 23 |
| Dyspnoea | Yes | 5 (9·5%) | 8 (15%) | 5 (12%) | 6 (21%) |
| | No | 47 | 44 | 37 | 23 |
| Anorexia | Yes | 5 (10%) | 13 (22%) | 9 (23%) | 9 (28%) |
| | No | 44 | 47 | 31 | 23 |
| Weight loss | Yes | 13 (25%) | 27 (41%) | 16 (42%) | 15 (45%) |
| | No | 40 | 39 | 22 | 18 |
| WBC $\geqslant$ 15,000 | | 2 (4%) | 7 (13%) | 2 (5%) | 3 (10%) |
| < 15,000 | | 50 | 47 | 37 | 26 |
| Hb. $\geqslant$ 11·4 g. | | 44 (84%) | 42 (75%) | 31 (76%) | 17 (59%) |
| < 11·4 g. | | 8 | 14 | 10 | 12 |
| Temperature $\geqslant$ 37·2° C | | 15 (45%) | 25 (64%) | 17 (57%) | 18 (82%) |
| < 37·2° C | | 18 | 14 | 13 | 4 |

survival found with the presence or absence of some of the more significant symptoms. A total lack of symptoms gave a mean and median survival in the Rye groups as shown in Table VIII.

TABLE VIII.—*Mean and Median Survival Times with Total Lack of Symptoms*

| Histological type | No. of patients | Mean survival (months) | Median survival (months) |
|---|---|---|---|
| Lymphocytic predominance | 23 | 112 | 125 |
| Nodular sclerosis | 13 | 96 | 84 |
| Mixed cellularity | 12 | 58 | 39 |
| Lymphocytic depletion | 7 | 95 | 60 |

It is noteworthy that in the lymphocytic depletion group the mean survival is 95 months signifying that histological interpretation alone is an insufficient guide to prognosis. It has been noted above that certain symptoms were of importance prognostically. If two symptoms in particular are selected—feeling unwell and lassitude, there is a striking similarity in the survival times irrespective of histological groupings as shown in Table IX.

TABLE IX.—*Mean and Median Survival Times with Lassitude and Feeling Unwell*

| Histological type | No. of patients | Mean survival (months) | Median survival (months) |
|---|---|---|---|
| Lymphocytic predominance | 16 | 67 | 60 |
| Nodular sclerosis | 11 | 56 | 55 |
| Mixed cellularity | 10 | 58 | 53 |
| Lymphocytic depletion | 7 | 57 | 29 |

9. *Relapses*.—The time interval between the first radiotherapy treatment and a further manifestation of disease was analysed. Of the initial 250 cases 67 (27%) did not relapse during the period of the study and remained disease free for more than 5 years. Eighty-nine patients (36%) died of their disease before any further radiotherapy could be given. This leaves 94 patients (38%) who had a further manifestation of disease after their first course of radiotherapy. The time interval to this recrudescence is illustrated in Fig. 7. It should be noted that 61% of patients who were to have a further manifestation of disease did so within 1 year and 98% within 5 years. In keeping with the clinical finding of a variable rate of progression of disease in different patients it was noted that in those in whom the disease recrudesced early (0–5 months after the first treatment) the median survival was only 46 months; in those where this took place later (6–11 months) the median survival was 62 months.

Thirty-one per cent of the 250 total cases had nodular sclerosing Hodgkin's disease but 49% of those that relapsed had this histological variety suggesting

FIG. 7.

that this group is more difficult to control by radiotherapy. Patients who died before a further manifestation of their disease could be treated are excluded. The percentage of relapses in the different histological groups is shown in Table X.

TABLE X.—*The Relationship of Histological Type to Relapses*

| | Total no. of cases | Relapsed | % |
|---|---|---|---|
| Lymphocytic predominance | 72 | 12 | 19 |
| Nodular sclerosis | 77 | 46 | 60 |
| Mixed cellularity | 62 | 22 | 35 |
| Lymphocytic depletion | 39 | 14 | 36 |

10. *Spread of disease.*—It has been suggested that Hodgkin's disease spreads in a largely predictable and orderly manner from one lymph node group to adjacent nodal areas (Rosenberg and Kaplan, 1966). A study was made of our 250 patients to determine to what extent the observed spread agreed with this concept of orderly progression. It should be remembered that the mediastinum and para-aortic regions are relatively silent areas where only gross disease will manifest itself clinically. Lymphangiography and mediastinal tomography were not in regular use at the time these cases presented.

An assessment was made of the most likely transition from one nodal area to another. For the purpose of this study it was assumed that all involved nodes resulted directly or indirectly from spread from the original primary, and that disease in a node detected on a certain occasion could have spread from any other involved nodal area with equal probability. Thus if *n* involved nodes were present on an occasion the probability of disease in a newly involved node having spread from any other node is $1/n$. These " transition probabilities " were calculated for each patient according to the nodes involved on various occasions during the course of the disease. The " probabilities " were then summed for all the patients. The sums would contain terms from transitions which do not occur but it was hoped that the transitions which occur most frequently would noticeably increase the sum of the " transition probabilities " for which they occur.

The " probability " of an involved node in one section causing disease in any other section was expressed as a percentage of the total numbers of transitions.

The most interesting and noticeable transitions are illustrated in Table XI.

TABLE XI.—*Spread of Disease*

| Primary | Secondary | % |
|---|---|---|
| Right neck | Left neck | 29 |
| | Mediastinum | 17 |
| | Right axilla | 16 |
| | Left axilla | 15 |
| Left neck | Left axilla | 19 |
| | Mediastinum | 16 |
| | Right axilla | 14 |
| Mediastinum | Left neck | 27 |
| | Right neck | 21 |
| Right axilla | Left axilla | 31 |
| | Right neck | 18 |
| Left axilla | Right axilla | 25 |
| | Left neck | 22 |
| Para-aortics | Left groin | 23 |
| | Right groin | 15 |
| Right groin | Left groin | 27 |
| | Left axilla | 15 |
| Left groin | Right groin | 29 |

It may be significant that disease in the axillae spreads to the mediastinum in only 6% of cases. This could be accounted for by the late detection of disease in the mediastinum. Mediastinal disease, however, progressed both to the right axilla (13%) and to the left axilla (12%). The proportion of transitions between axillae is higher than expected on anatomical grounds and has been noted by Hutchinson (1970). It is impossible to say whether or not the mediastinum was involved *en route*. We have not been able to confirm the recent anatomical correlation between the left side of neck and the para-aortic nodes as the latter area being clinically silent did not score highly in our investigation.

TREATMENT

All patients were treated initially by irradiation. The treatment employed during the period of this study was radiotherapy to the nodal areas involved. The type of radiation used was either 60 Cobalt or 250 kV x-rays. The nodal dose employed did not usually exceed 3500 rad in 3·5–4 weeks. Prophylactic irradiation of the mediastinum advocated by Peters and Middlemiss (1958) was adopted towards the end of this series. The mediastinum was irradiated when disease presented in the neck or axilla in the absence of mediastinal or hilar disease. Sixty-seven patients were treated in this manner and, although no statistically significant difference could be found using the $\chi^2$ test, there was a noticeable difference in projected survival time using the actuarial method, *e.g.* 69% 5-year survival with prophylactic mediastinal irradiation as compared with 49% surviving 5 years without this treatment as shown in Table XII.

TABLE XII.—*Survival Rates* for Patients With and Without Prophylactic Radiotherapy of the Mediastinum*

| Survival time (years) | Survival rate with mediastinal irradiation | Survival rate without mediastinal irradiation |
|---|---|---|
| 1 | 97% | 88% |
| 2 | 88% | 74% |
| 5 | 69% | 49% |
| 7 | 57% | 38% |
| 10 | 52% | 31% |
| 15 | 45% | 23% |

* Calculated by the actuarial method.

Extended field radiation or diagnostic laparotomy and splenectomy as recently advocated by Glatstein *et al.* (1970) were not used at the time of this study and lymphangiography and mediastinal tomography were not in routine use.

No mention will be made during the discussion of the effect of chemotherapy on survival. This was employed mainly for generalized disease (Stage IV) and usually only single agents were used.

DISCUSSION

A reading of the literature of Hodgkin's disease since the Rye classification came into being reveals certain very disturbing features. Firstly, there is the very wide variation from series to series in the incidence of nodular sclerosis mentioned earlier. Secondly, the usefulness of any classification must be decreased when a team of pathologists cannot agree amongst themselves on a series of cases as indicated by Keller *et al.* (1968). Thirdly, such differences must account, in part at any rate, for the very wide differences in survival times in different series.

In the present series we found the Rye classification moderately easy to use although certain recurring difficulties were encountered. Problems connected with poor quality histological sections have already been mentioned. In a number of cases we had difficulty in deciding whether a case should be allocated to the lymphocytic predominance group or to mixed cellularity. In others minimal collagen and doubtful lacuna cells created difficulty with the nodular sclerosing type.

Hitherto the major prognostic factors in Hodgkin's disease have been clinical staging and histology. Clinical staging lays stress on the importance of the diaphragm as an anatomical division (Table II). Although differences in 5-year survival figures have been detected in these different clinical stages (Smithers, 1969), Cross and Dixon (1971) showed that this anatomical consideration did not have the same importance as symptoms and histological grades. The survival figures (54% at 5 years) of our 250 patients seen between the years 1954 and 1966 are comparable with other series of that time (Peters and Middlemiss, 1958; Smithers, 1969).

Little attention has been given to individual clinical features alone, or in combination with histological grades. An attempt has been made in this paper to correlate these factors. We have been able to demonstrate that pruritus has no prognostic value except in the lymphocytic predominant cases. The important factors are night sweats, anorexia, lassitude, feeling unwell and weight loss. In addition a high white blood count (above 15,000), a low Hb. (below 11·4 g) and temperature recording on first attendance of 37·2° C or above carried a poor prognosis. Splenic enlargement on presentation also adversely affected prognosis. These observations tend to show that there are circumstances when the presence or absence of certain symptoms are of greater significance than histological grading. It is advocated that when an attempt is made to assess the prognosis in Hodgkin's disease, symptomatology should always be correlated with histology if an accurate guide to prognosis is sought.

We are indebted to our clinical colleagues at Westminster Hospital, in the Royal Army Medical Corps and in the Royal Air Force for referring cases. We acknowledge the secretarial help given by Mrs M. Dixon, Mrs M. Taor and Mrs A. Leaver. We thank the Department of Medical Photography, Westminster Hospital.

### REFERENCES

Berkson, J. & Gage, R. P. (1950) Calculation of Survival Rates of Cancer. *Proc. Staff Meet. Mayo Clin.*, **25**, 270.

Cross, R. M. (1968) A Clinico-pathological Study of Nodular Sclerosing Hodgkin's Disease. *J. clin. Path.*, **21**, 303.

Cross, R. M. (1969) Hodgkin's Disease: Histological Classification and Diagnosis. *J. clin. Path.*, **22**, 165.

Cross, R. M. & Dixon, F. W. P. (1971) A Combined Clinical and Histological Assessment of Survival of Patients with Hodgkin's Disease. *J. clin. Path.*, **24**, 385.

Easson, E. C. (1966) Long Term Results of Radical Radiotherapy in Hodgkin's Disease. *Cancer Res.*, **26**, 1244.

Franssila, K. O., Kalima, T. V. & Voutilainen, A. (1967) Histologic Classification of Hodgkin's Disease. *Cancer, N.Y.*, **20**, 1594.

Fuller, L. M., Gamble, J. F., Shullenberger, C. C., Butler, J. J. & Gehan, E. A. (1971) Hodgkin's Disease Treated with Regional Radiation. *Radiology*, **98**, 641.

Glatstein, E., Trueblood, H. W., Enright, L. P., Rosenberg, S. A. & Kaplan, H. S. (1970) Surgical Staging of Abdominal Involvement in Unselected Patients with Hodgkin's Disease. *Radiology*, **97**, 425.

Hutchinson, G. B. (1970) Anatomic Patterns by Histologic Type of Localised Hodgkin's Disease. In *Third International Congress of Lymphology, Brussels.*

Johnson, R. E., Thomas, L. B., Sniederman, M., Glenn, D. W., Faw, F. & Hafermann, M. D. (1970) Preliminary Experience with Total Nodal Irradiation in Hodgkin's Disease. *Radiology*, **96**, 603.

Kadin, M. E., Glatstein, E. & Dorfman, R. F. (1971) Clinico-pathologic Studies of 117 Untreated Patients Subjected to Laparotomy for the Staging of Hodgkin's Disease. *Cancer, N.Y.*, **27**, 1277.

Keller, A. R., Kaplan, H. S., Lukes, R. J. & Rappaport, H. (1968) Correlation of Histopathology with Other Prognostic Indicators in Hodgkin's Disease. *Cancer, N.Y.*, **22**, 487.

Lukes, R. J., Craver, L. F., Rappaport, H. & Rubin, P. (1966*a*) Report of the Nomenclature Committee. *Cancer Res.*, **26**, 1311.

Lukes, R. J., Butler, J. J. & Hicks, E. B. (1966*b*) Natural History of Hodgkin's Disease as Related to its Pathologic Picture. *Cancer, N.Y.*, **19**, 317.

Lumb, G. D. & Newton, K. A. (1957) Prognosis in Tumours of Lymphoid Tissue. *Cancer, N.Y.*, **10**, 976.

Peters, M. V. & Middlemiss, K. C. H. (1958) A Study of Hodgkin's Disease Treated by Irradiation. *Am. J. Roentg.*, **79**, 114.

Rosenberg, S. A. & Kaplan, H. S. (1966) Evidence for an Orderly Progression in the Spread of Hodgkin's Disease. *Cancer Res.*, **26**, 1225.

Rosenberg, S. A., Boiron, M., DeVita, V., Johnson, R. E., Lee, B. J., Ultmann, J. E. & Viamonte, M. (1971) Report of the Committee on Hodgkin's Disease Staging Procedures. *Cancer Res.*, **31**, 1862.

Smithers, D. W. (1969) Factors Influencing Survival in Patients with Hodgkin's Disease. *Radiology*, **20**, 124.

Strüm, S. B. & Rappaport, H. (1971) Interrelationships of the Histologic Types of Hodgkin's Disease. *Archs Path.*, **91**, 127.


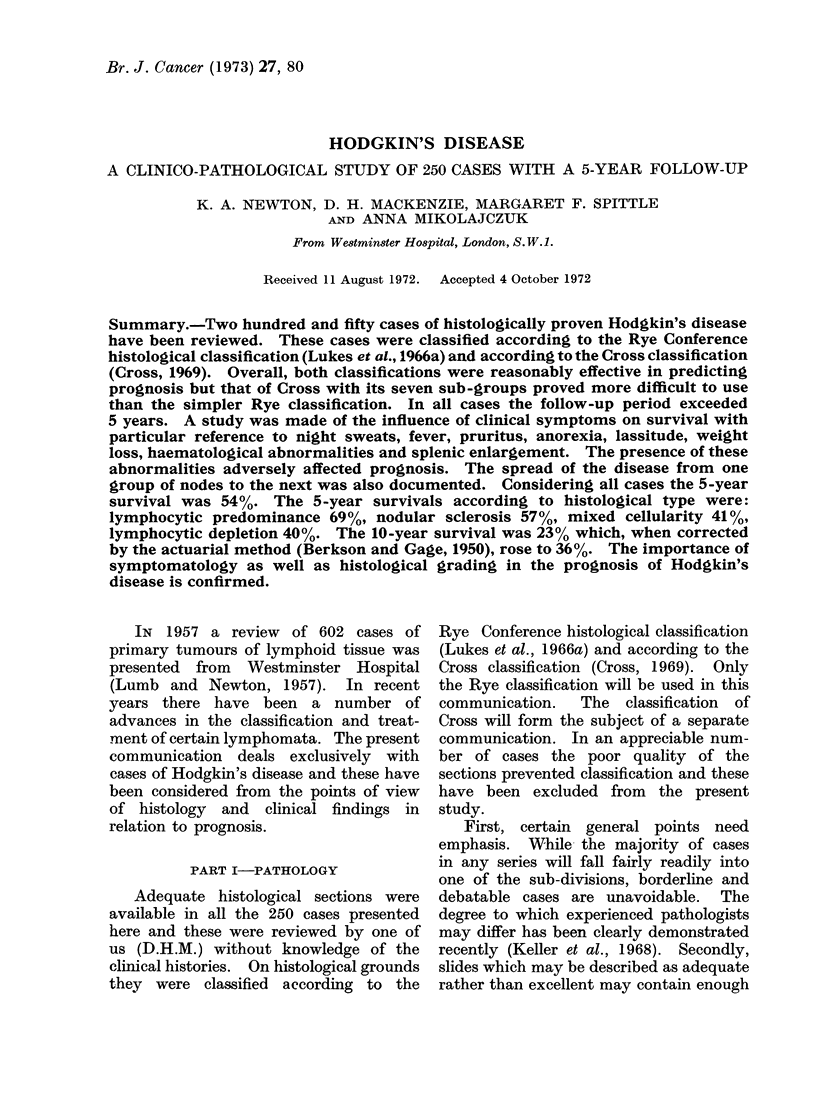

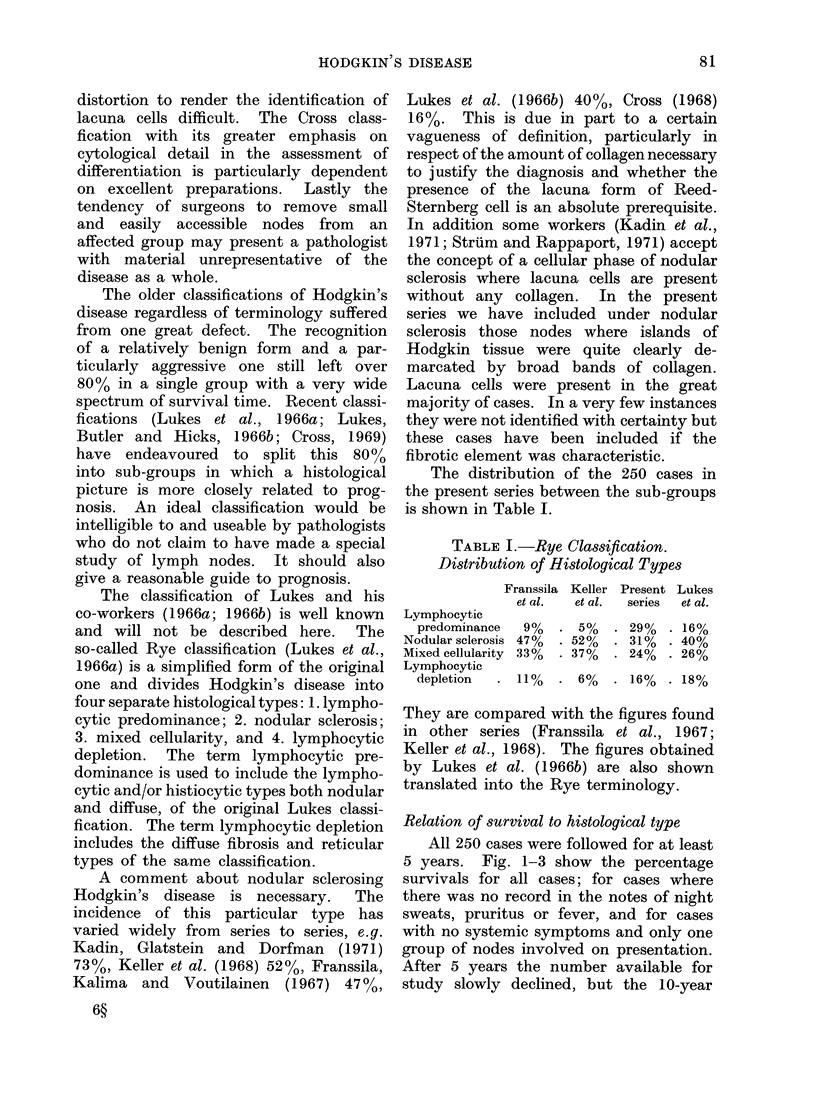

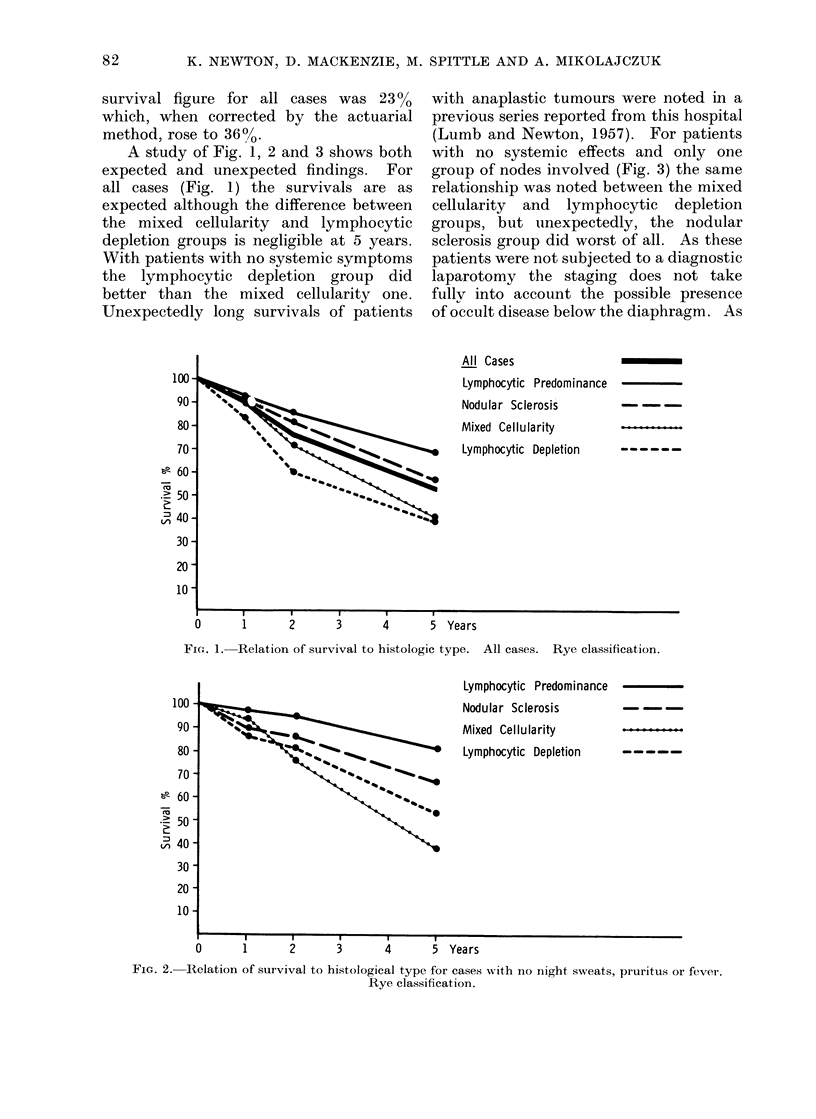

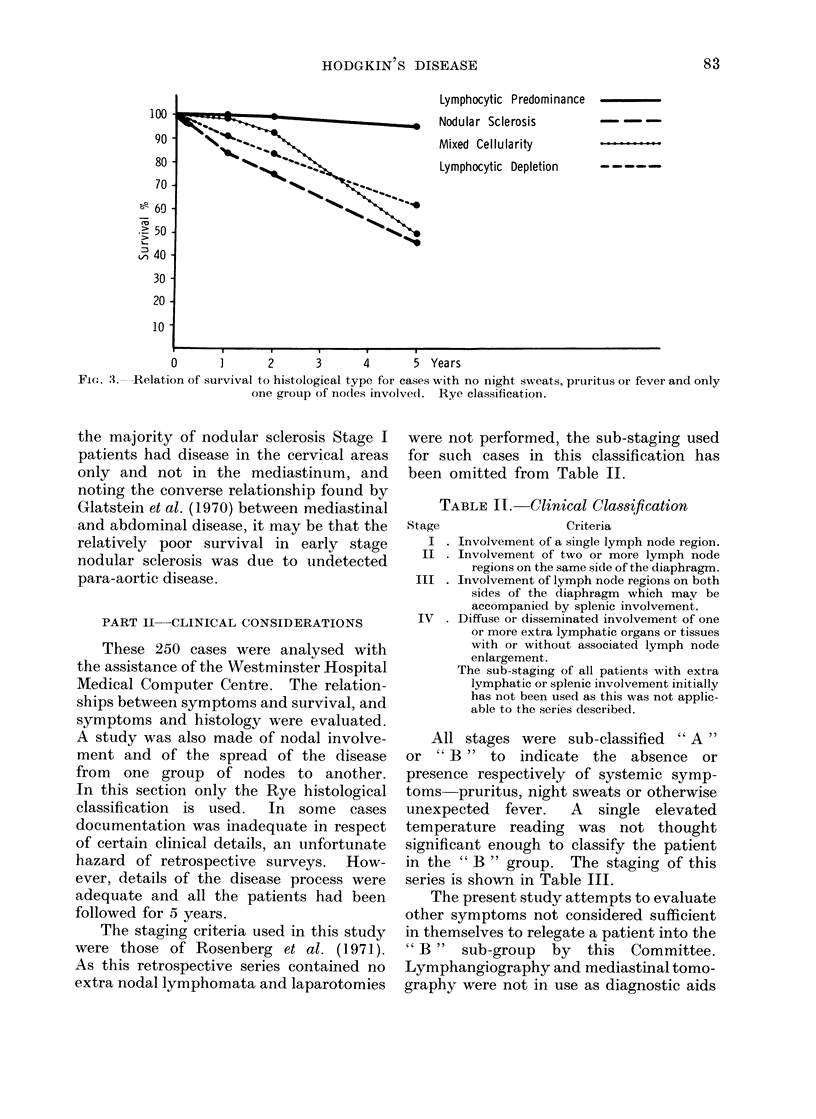

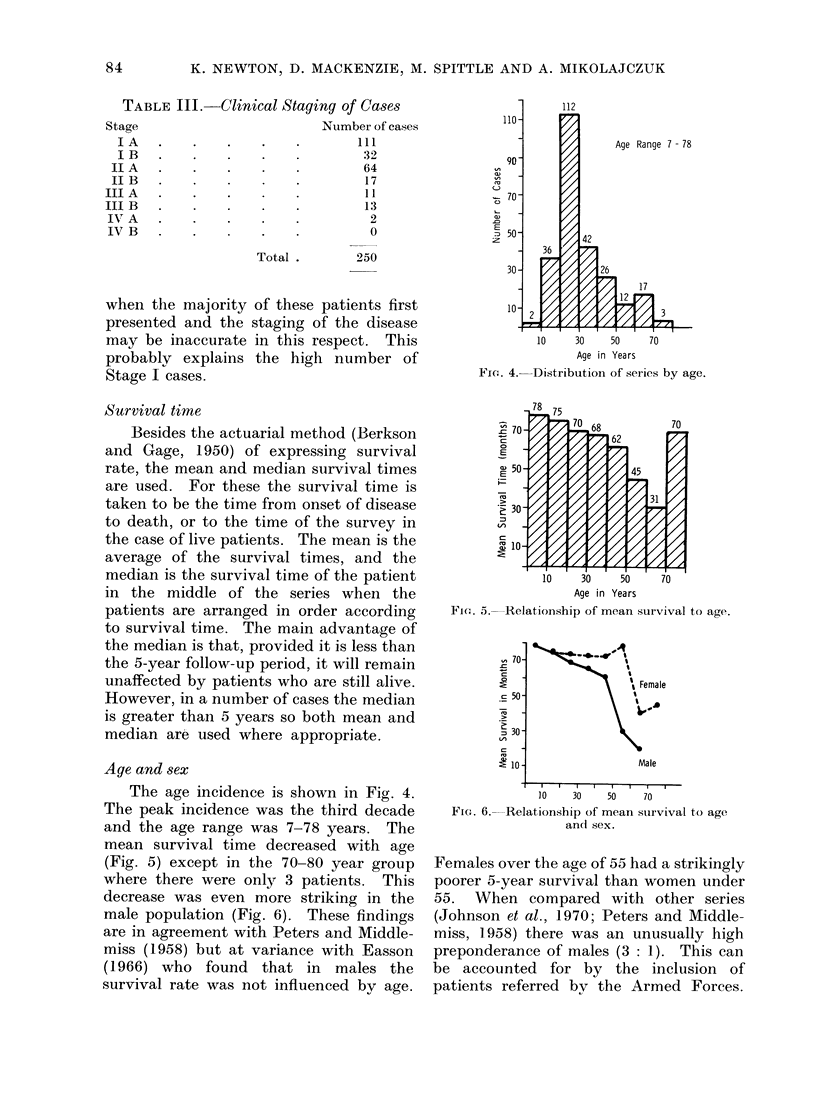

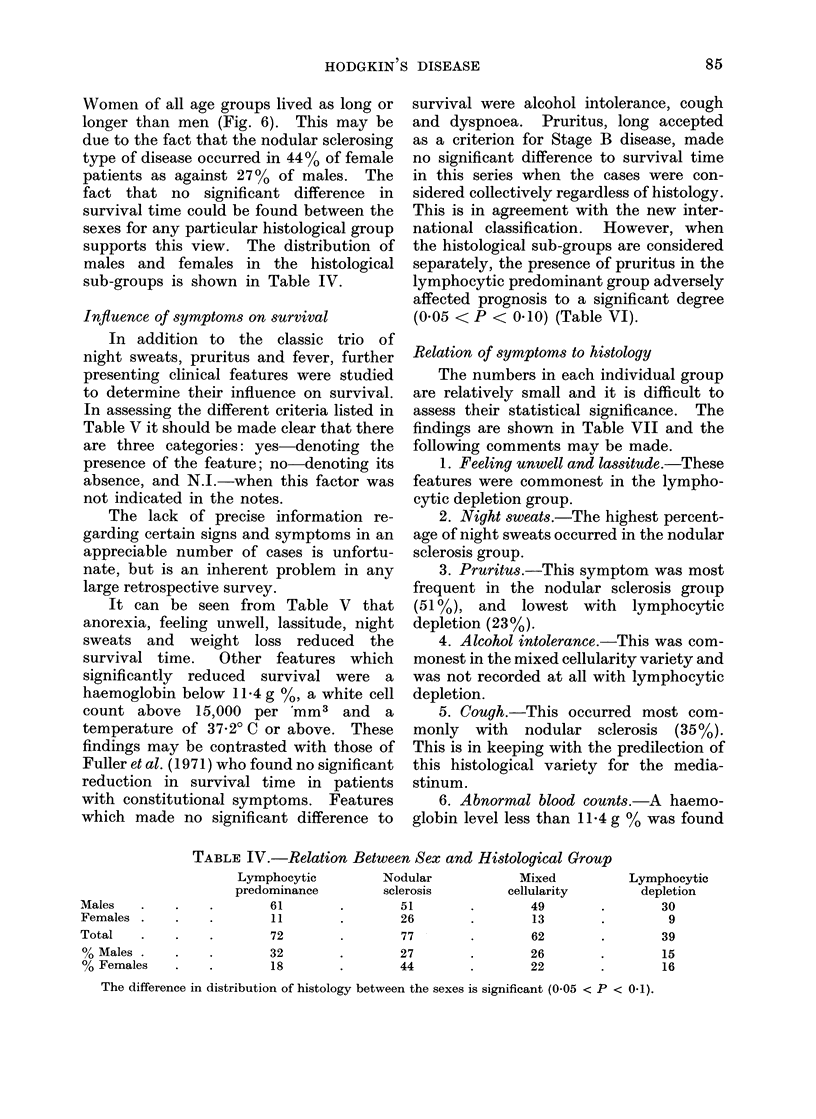

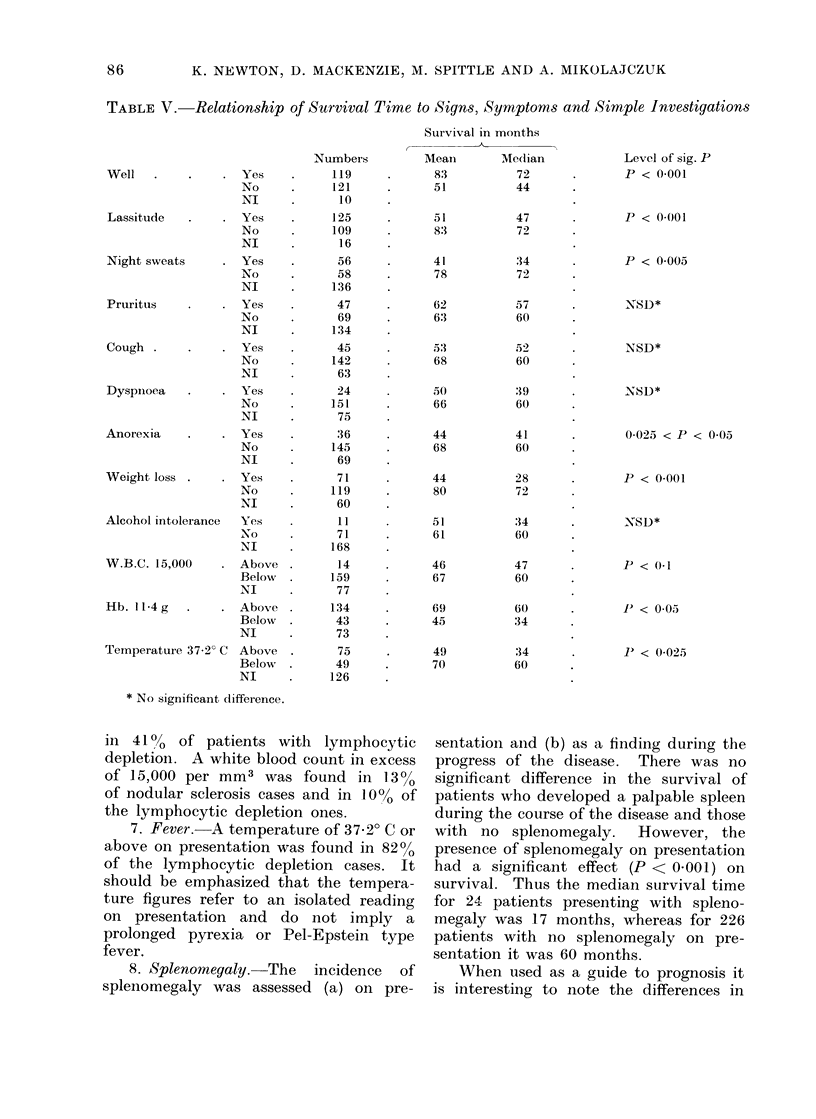

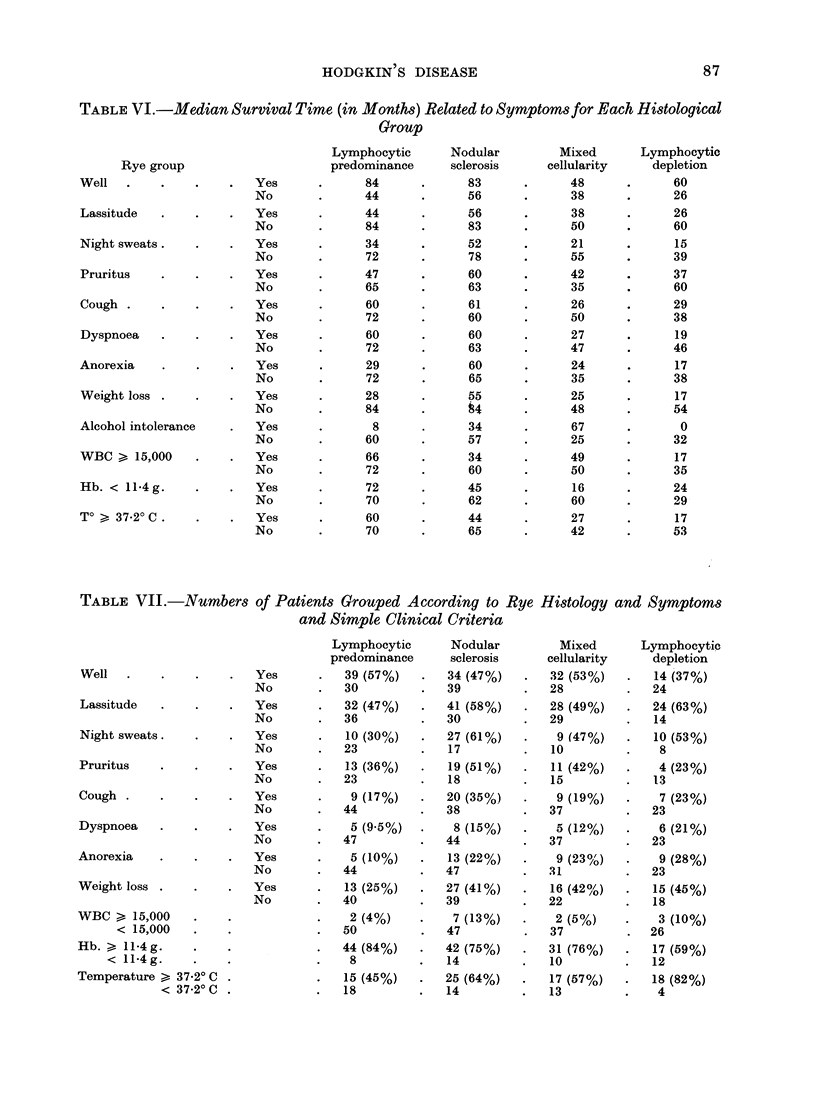

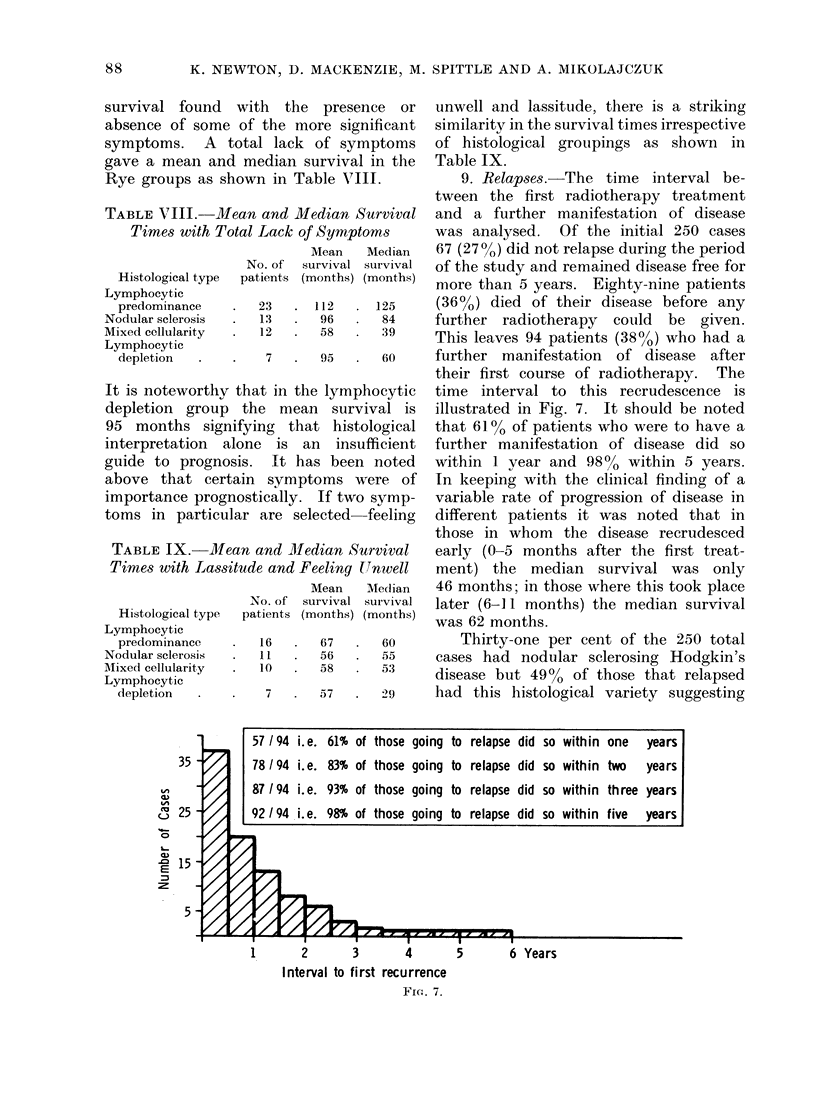

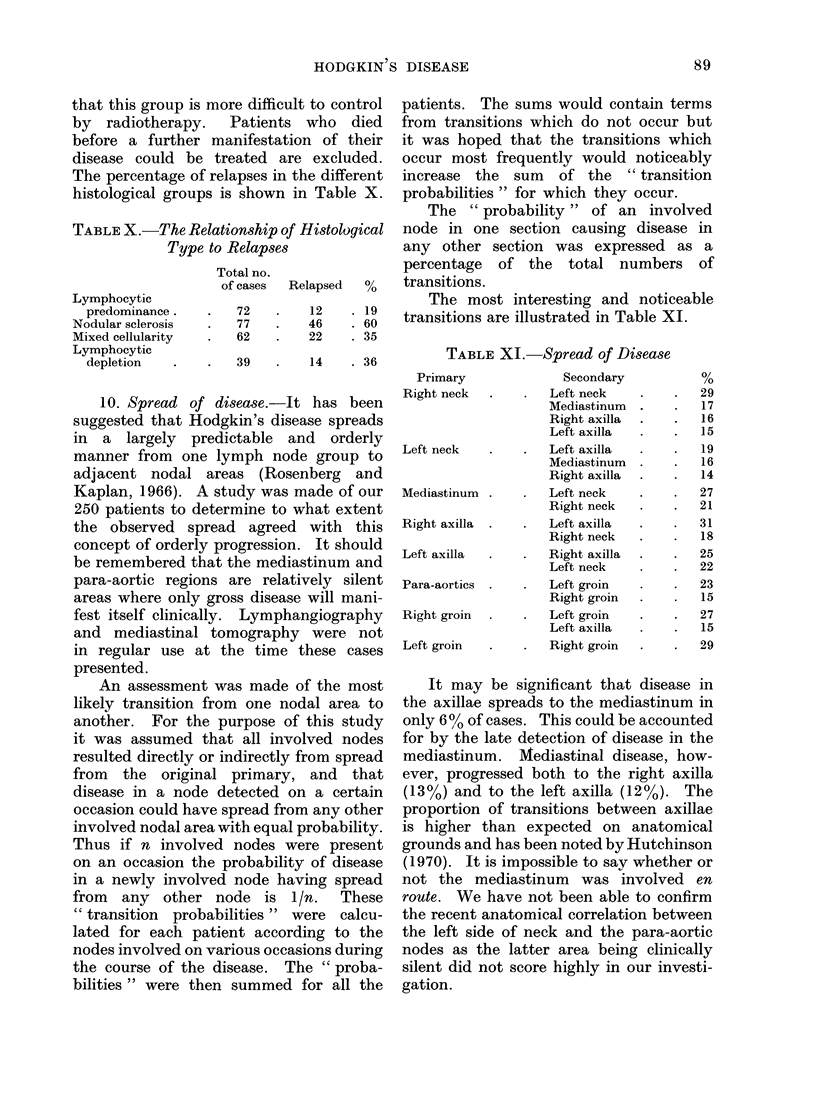

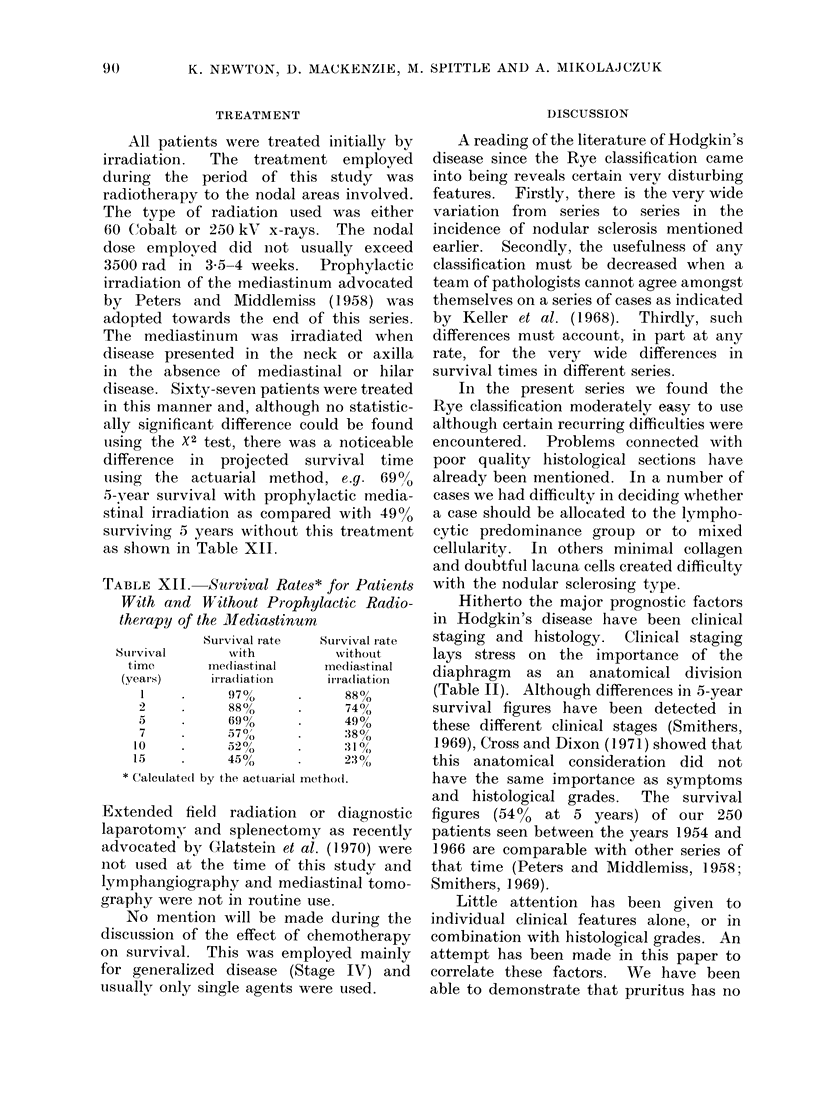

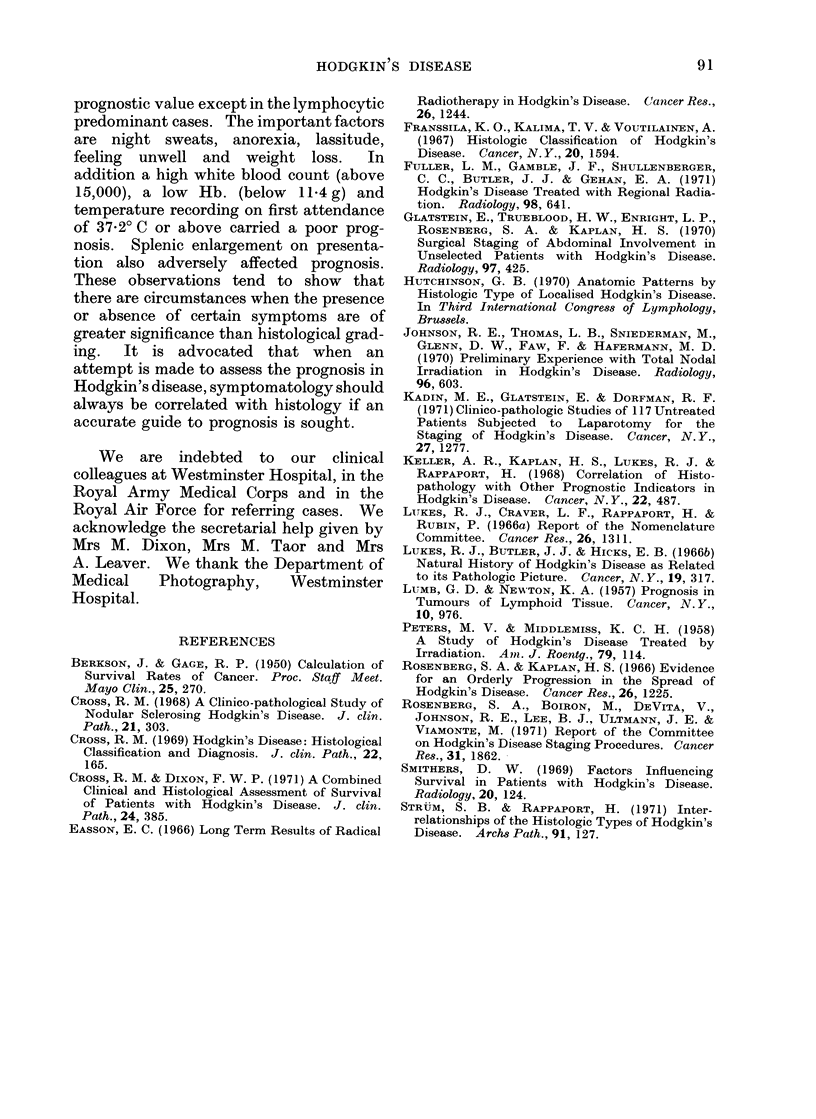


## References

[OCR_01446] BERKSON J., GAGE R. P. (1950). Calculation of survival rates for cancer.. Proc Staff Meet Mayo Clin.

[OCR_01451] Cross R. M. (1968). A clinicopathological study of nodular sclerosing Hodgkin's disease.. J Clin Pathol.

[OCR_01461] Cross R. M., Dixon F. W. (1971). A combined clinical and histological assessment of survival of patients with Hodgkin's disease.. J Clin Pathol.

[OCR_01456] Cross R. M. (1969). Hodgkin's disease: histological classification and diagnosis.. J Clin Pathol.

[OCR_01467] Easson E. C. (1966). Long-term results of radical radiotherapy in Hodgkin's disease.. Cancer Res.

[OCR_01472] Franssila K. O., Kalima T. V., Voutlainen A. (1967). Histologic classification of Hodgkin's disease.. Cancer.

[OCR_01477] Fuller L. M., Gamble J. F., Shullenberger C. C., Butler J. J., Gehan E. A. (1971). Prognostic factors in localized Hodgkin's disease treated with regional radiation. Clinical presentation and specific histology.. Radiology.

[OCR_01483] Glatstein E., Trueblood H. W., Enright L. P., Rosenberg S. A., Kaplan H. S. (1970). Surgical staging of abdominal involvement in unselected patients with Hodgkin's disease.. Radiology.

[OCR_01496] Johnson R. E., Thomas L. B., Schneiderman M., Glenn D. W., Faw F., Hafermann M. D. (1970). Preliminary experience with total nodal irradiation in Hodgkin's disease.. Radiology.

[OCR_01503] Kadin M. E., Glatstein E., Dorfman R. F. (1971). Clinicopathologic studies of 117 untreated patients subjected to laparotomy for the staging of Hodgkin's disease.. Cancer.

[OCR_01510] Keller A. R., Kaplan H. S., Lukes R. J., Rappaport H. (1968). Correlation of histopathology with other prognostic indicators in Hodgkin's disease.. Cancer.

[OCR_01525] LUMB G., NEWTON K. A. (1957). Prognosis in tumors of lymphoid tissue; an analysis of 602 cases.. Cancer.

[OCR_01530] PETERS M. V., MIDDLEMISS K. C. (1958). A study of Hodgkin's disease treated by irradiation.. Am J Roentgenol Radium Ther Nucl Med.

[OCR_01540] Rosenberg S. A., Boiron M., DeVita V. T., Johnson R. E., Lee B. J., Ultmann J. E., Viamonte M. (1971). Report of the Committee on Hodgkin's Disease Staging Procedures.. Cancer Res.

[OCR_01535] Rosenberg S. A., Kaplan H. S. (1966). Evidence for an orderly progression in the spread of Hodgkin's disease.. Cancer Res.

[OCR_01547] Smithers D. W. (1969). Factors influencing survival in patients with Hodgkin's disease.. Clin Radiol.

[OCR_01552] Strum S. B., Rappaport H. (1971). Interrelations of the histologic types of Hodgkin's disease.. Arch Pathol.

